# Reconstruction of the esophagojejunostomy by double stapling method using EEA™ OrVil™ in laparoscopic total gastrectomy and proximal gastrectomy

**DOI:** 10.1186/1477-7819-9-55

**Published:** 2011-05-20

**Authors:** Noriyuki Hirahara, Hiroyuki Monma, Yoshihide Shimojo, Takeshi Matsubara, Ryoji Hyakudomi, Seiji Yano, Tsuneo Tanaka

**Affiliations:** 1 Department of Digestive and General Surgery, Shimane University School of Medicine, 89-1 Enya-cho, Izumo, Shimane 693-8501, Japan

**Keywords:** Esophagojejunostomy, Double stapling method, EEA™ OrVil™

## Abstract

Here we report the method of anastomosis based on double stapling technique (hereinafter, DST) using a trans-oral anvil delivery system (EEATM OrVilTM) for reconstructing the esophagus and lifted jejunum following laparoscopic total gastrectomy or proximal gastric resection.

As a basic technique, laparoscopic total gastrectomy employed Roux-en-Y reconstruction, laparoscopic proximal gastrectomy employed double tract reconstruction, and end-to-side anastomosis was used for the cut-off stump of the esophagus and lifted jejunum.

We used EEATM OrVilTM as a device that permitted mechanical purse-string suture similarly to conventional EEA, and endo-Surgitie.

After the gastric lymph node dissection, the esophagus was cut off using an automated stapler. EEATM OrVilTM was orally and slowly inserted from the valve tip, and a small hole was created at the tip of the obliquely cut-off stump with scissors to let the valve tip pass through. Yarn was cut to disconnect the anvil from a tube and the anvil head was retained in the esophagus.

The end-Surgitie was inserted at the right subcostal margin, and after the looped-shaped thread was wrapped around the esophageal stump opening, assisting Maryland forceps inserted at the left subcostal and left abdomen were used to grasp the left and right esophageal stump. The surgeon inserted anvil grasping forceps into the right abdomen, and after grasping the esophagus with the forceps, tightened the end Surgitie, thereby completing the purse-string suture on the esophageal stump.

The main unit of the automated stapler was inserted from the cut-off stump of the lifted jejunum, and a trocar was made to pass through. To prevent dropout of the small intestines from the automated stapler, the automated stapler and the lifted jejunum were fastened with silk thread, the abdomen was again inflated, and the lifted jejunum was led into the abdominal cavity.

When it was confirmed that the automated stapler and center rod were made completely linear, the anvil and the main unit were connected with each other and firing was carried out. Then, DST-based anastomosis was completed with no dog-ear.

The method may facilitate safe laparoscopic anastomosis between the esophagus and reconstructed intestine. This is also considered to serve as a useful anastomosis technique for upper levels of the esophagus in laparotomy.

## Background

Previously it has reported that safe and easy way of conducting anastomosis between the esophagus and digestive tract following total or proximal gastrectomy by the hemi-double stapling technique using EEA™ OrVil^TM1 ^[1]. In this technique there was always a dog ear, and even though we were able to maintain blood flow, we were unable to resolve the weak point.

But we sought to improve this technique, we identified double stapling method for esophagojejunostomy with no overlapping spots of the stapler in this report.

## Methods

### Subjects

As the basic procedure, early gastric cancer with lesions localized at the upper part of the stomach employed laparoscopic proximal excision and double-tract reconstruction while early gastric cancer with lesions spreading in the upper and middle regions employed laparoscopic total gastrectomy and Roux-en-Y reconstruction. End-to-side anastomosis was used for reconstructing the removed stump of the esophagus and lifted jejunum.

### Devices Used

**Figure 1:**
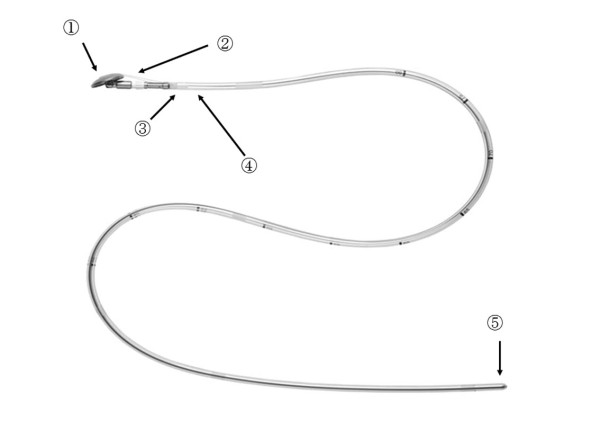
**Components of EEA™ OrVil™**. 1. Anvil head. 2. Anvil holding yarn (No. 1 polyester yarn). 3. Colored plastic section. 4. Center rod. 5. Valve tip.

EEA™ OrVil™ is a device that permits mechanical purse-string suture. An anvil head with a diameter of 21 or 25 mm is fastened in a tilted state at about 170 degrees to a tube as long as about 95 mm via No. 1 polyester yarn with a white plastic connector. The tube is calibrated in 5-cm increments starting with the anvil head. The tip of the tube is called a valve tip. Purse-string suture is enabled by connecting the center rod of the anvil head and the main unit of the automated stapler, and conducting firing(Figure 1).

We used Surgitie™ for purse-string suture of esophagus.

### Posture

In general cases, for the basic operation, the patient was kept in a spine position with his/her legs opened. A scopist stood between the patient's legs. An operator stood on the right-hand side and a primary assistant stood on the left-hand side of the patient.

### Site of insertion of a trocar

A 12-mm-long trocar was inserted below the umbilicus as a port for laparoscope. Trocars with different sizes were inserted as working ports under abdominal inflation with 8 to 10 mmHg: a 5-mm-long trocar under the right lumbocostal arch; 12-mm-long for the right abdomen; 12-mm-long under the left lumbocostal arch; and 5-mm-long for the left abdomen. The lateroabdominal trocars were placed slightly inward from the right and left lumbocostal arches: trocars formed an inverted trapezium.

### Removal of tissue samples

**Figure 2:**
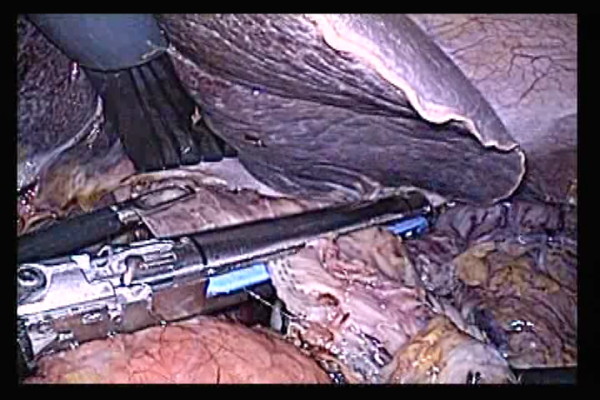
**The esophagus was cut off obliquely to the long axis with the automated stapler inserted from the right abdomen**.

Following dissection of the gastric lymph nodes, an automated stapler was inserted via a trocar of the right abdomen, and tissues of the esophagus were cut off (Figure 2).

A 7-cm-long small abdominal incision was created at the midline slightly caudal from the ensiform process of the epigastric region. Samples were led outside the abdominal cavity.

For total gastrectomy, the duodenum was cut off immediately under the pylorus. For proximal gastrectomy, a sufficient distance from the open end was secured so as not to leave any tumor remnants, and cut-off was performed with an automated stapler at the gastric body.

## Esophago-jejunostomy

### Placement of an anvil head within the esophagus

EEA™ OrVil™ was orally inserted slowly from the valve tip until the valve tip reached the open end of the esophagus.

**Figure 3:**
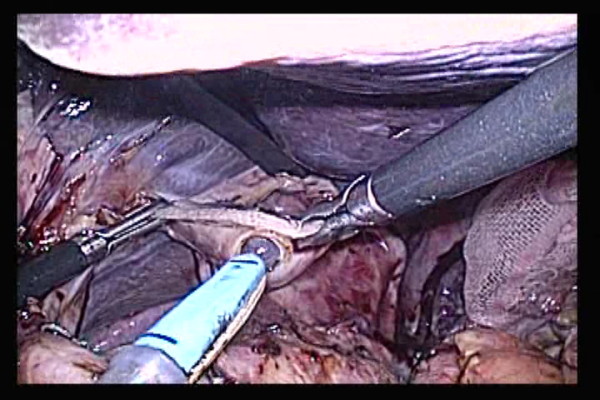
**A small hole was created at the tip of the open end of the esophagus obliquely cut off to the long axis**.

A small hole was created with electric scissors at the tensed site while tension was confirmed. The valve tip was made to pass through (Figure 3).

**Figure 4:**
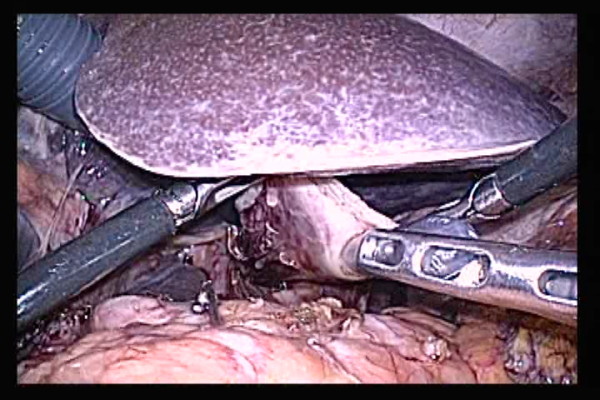
**A tube was made to pass through from a small hole at the tip of the open end of the esophagus**.

Straight grasping forceps were inserted via a trocar at the left abdomen (Figure 4, a). A tube was led outside the abdominal cavity while the valve tip at a small hole at the open end of the esophagus was being grasped. The cuffs of the endotracheal intubation tube tend to cause resistance during transit. To alleviate this resistance, the throat cavity was widened during transit through the larynx, and the cuffs were deflated. When the tube was pulled further, the anvil was led from the open end of the esophagus into the abdominal cavity. Then, the grasping notch of the center rod was securely grasped with anvil straight grasping forceps. The anvil head and the tube were connected with two pieces of No. 1 polyester yarn, which were cut to disconnect the anvil and place the anvil head within the esophagus.

### Purse-String Suture of the Esophageal Stump

**Figure 5:**
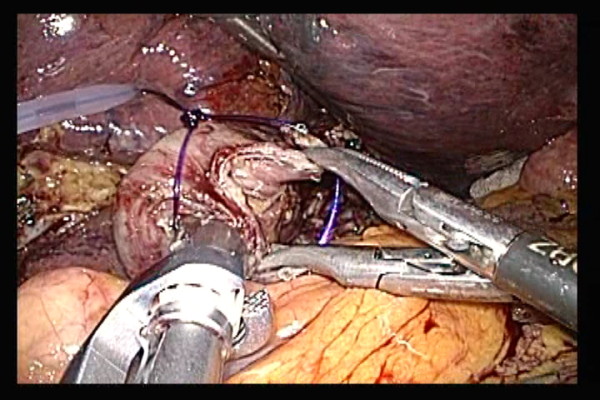
**The looped-shaped thread was wrapped around the esophageal stump opening, assisting Maryland forceps were used to grasp the left and right esophageal stump**.

**Figure 6:**
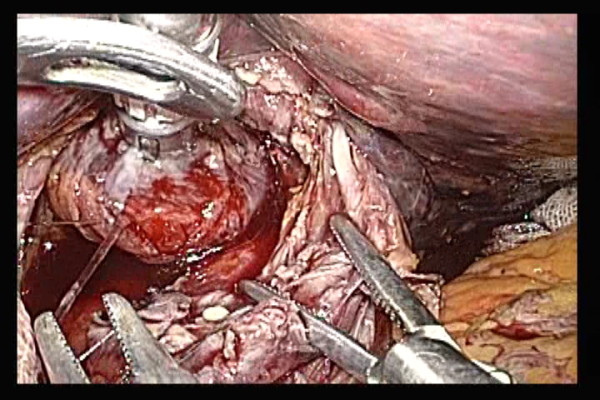
**The surgeon tightened the Surgitie™, thereby completing the purse-string suture on the esophageal stump**.

The Surgitie™ was inserted at the right subcostal margin, and after the looped-shaped thread was wrapped around the esophageal stump opening, assisting Maryland forceps inserted at the left subcostal and left abdomen were used to grasp the left and right esophageal stump (Figure 5). The surgeon inserted anvil grasping forceps into the right abdomen, and after grasping the esophagus with the forceps, tightened the Surgitie™, thereby completing the purse-string suture on the esophageal stump (Figure 6).

### Preparation of lifted jejunum

The jejunum 20 cm away from the ligament of Treitz was led from a small abdominal incision to outside the abdominal cavity and also cut off with an automated stapler. An automated stapler was inserted from the open end of the lifted jejunum to let a trocar pass through. Then, the main unit of the automated stapler and the lifted jejunum were fastened with silk thread to prevent dropout of the small intestines from the automated stapler. The abdomen was again inflated and the lifted jejunum was led into the abdominal cavity.

### Connection with anvil, and anastomosis

The anvil and the main unit were connected after it was confirmed that an automated stapler and the center rod were made fully linear. Firing then completed the anastomosis.

**Figure 7:**
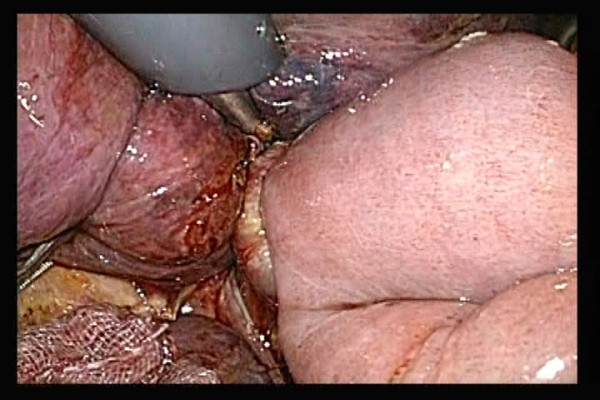
**The anastomosis site was checked in multiple directions to make sure the jejunum was not caught in the anastomosis site**.

The inlet of the automated stapler at the open end of the lifted jejunum was closed with an automated stapler inserted via a trocar of the right abdomen. Then, anastomosis between the esophagus and jejunum was completed(Figure 7).

## Discussion

We have been reported on the use of the EEA™ OrVil™ stapler in end-to-side anastomosis for esophagojejunostomy with the hemi-double stapling procedure, although there was some minor leakage experienced during the 35^th ^case, we sought to improve this technique [2].

For rectal cancer, reconstruction during the low anterior resection is generally performed with the double stapling technique, with ruptured sutures being reported in 2.6-17% of cases [3-6]. The existence of dog ears at the site of ruptured sutures could not be confirmed in all cases, but when dog ears formed the weak point, it was necessary to consider the blood flow around the anastomosis site [7]. In our report of the hemi double stapling technique there was always a dog ear, and even though we were able to maintain blood flow, we were unable to resolve the weak point. Moreover, an advanced technique is necessary when a purse-string suture with an anvil insertion is used as the suturing technique at the esophageal stump, and due to its cumbersome nature, various measures have been devised. To perform a resection similar to a laparotomy, we have developed the easily performed complete double stapling method.

On the anvil placed at the remaining esophageal side, Surgitie™ was used in addition to the purse-string suture. The stapler used at the time of resecting the esophageal stump became the stopper, and even if the purse-string suture was not inserted into esophageal stump, the ligature of the end Surgitie was sufficient to close the stump end without coming apart.

An anastomis for esophagojejunostomy usually requires EEA™φ 25 mm, but the EEA™ OrVil™φ 25 mm that we use--compared with the conventional EEA™ φ 25 mm--enlarged the external diameter from 25 mm to 25.6 mm. The diameter of the resection site also increased from 15 to 16.5 mm, and the surface area increased by about 21%. Consequently, the surplus esophageal stump created by using a purse-string suture makes the esophageal stump stapler easy to employ. Because we have no experience conducting anastomis with EEA™φ 21 mm, it is unclear whether the surplus esophageal wall can be stapled without undue effort, but in all 8 resections we have conducted using the EEA™ OrVil™φ25 mm, it was possible to staple along the stapler line completely.

Accordingly, the highly stressful interperitoneal suturing technique used by surgeons performing this microscopic esophagojejunostomy is unnecessary and has been made simple. However, further case studies must be assessed and monitored to test for safety and reliability in a randomized fashion.

## Competing interests

The authors declare that they have no competing interests.

## Authors' contributions

NH was the lead author and surgeon for all of the patients. HM gathered information and contributed to writing of the paper. YS and TM contributed patients and information on the patients. RH and SY were the co-surgeon on the cases. TT reviewed paper and technique of surgery.

All authors read and approved the final manuscript.

## Consent

Written informed consent was obtained from the patient for publication of this case report and any accompanying images. A copy of the written consent is available for review by the Editor-in-Chief of this jounal.
